# Patterns of cancer in Needle Hospital, Hargeisa, Somaliland from July 2022 to June 2023

**DOI:** 10.3332/ecancer.2025.1904

**Published:** 2025-05-13

**Authors:** Gebrekirstos Hagos, Nazik Hammad, Susannah Stanway, Verna Dnk Vanderpuye, Abdikani Yusuf, Tekleberhan Hailemariam, Osman Ahmed, Husein Jamac, Ubah Ahmed

**Affiliations:** 1 Needle Hospital, Hargeisa, 90203, Somaliland; 2 Saint Michael’s Hospital, University of Toronto, Toronto, M5B 1W8, Canada; 3 Royal Marsden Hospital, Sutton Surrey, SM2 5PT, UK; 4 National Center for Radiotherapy Oncology and Nuclear Medicine, Korle Bu Teaching Hospital, Accra, 0000, Ghana; 5 Dire Dawa University, Dire Dawa, 1362, Ethiopia; 6 University of Hargeisa, Hargeisa, Somaliland

**Keywords:** pattern of cancer, Needle Hospital, Somaliland

## Abstract

**Purpose:**

Globally, the incidence of and mortality from cancer is rapidly increasing and presents a barrier to increasing life expectancy. Based on regional and global trends, cancer incidence in Somaliland is expected to increase. Until recently, there was no dedicated cancer clinic in Somaliland. In July 2022, a medical oncology service was started in Needle Hospital, Hargeisa, Somaliland. This study reports on patterns of cancer with respect to patients’ region, age, gender, comorbidities, site and subsites of cancer, histology and stages.

**Patients and method:**

A retrospective study was conducted to determine the patterns of cancer among patients evaluated in the Needle Hospital cancer clinic from July 2022 to June 2023. Data were extracted from the cancer patient registration file and charts. Descriptive statistics were applied using the Statistical Package for the Social Sciences version 23.

**Results:**

A total of 232 cancer patients were evaluated during the study period. The median age was 60.0 years. More than half (56.5%) of the patients were female, with a female-to-male ratio of 1.3:1. Most of the patients (66.8%) came from Morodijeh, followed by Togdher (15.1%) and Awdal (5.2%) regions. The most common anatomic subsites of the cancers were breast, esophageal and prostate cancers, accounting for 15.9%, 8.2% and 7.3%, respectively. Based on histology, adenocarcinoma and squamous cell carcinoma accounted for 42.2% and 25%, respectively. Most patients presented at an advanced stage; stage IV cancer accounted for 44.4% and stage III cancers accounted for 29.30% of the total patients.

**Conclusion:**

Based on this study, cancer is one of the emerging health problems in Somaliland. Most patients presented at an advanced stage. Breast, esophageal and prostate cancers were the most commonly diagnosed cancers. Esophageal cancer, being a common finding, is disparate, so a study investigating the aetiology and biology of esophageal cancer in Somaliland is recommended. We also recommend establishing the National Cancer Control Plan, a national cancer registry and developing research capacity. Finally, to improve cancer outcomes, capacity building in diagnostic and treatment facilities and regional and international collaboration should also be prioritised.

## Introduction

Globally, the incidence and mortality of cancer are rapidly increasing, and it is one of the barriers to increasing life expectancy [1, 2]. The type and pattern of cancer vary by geographical region, people’s lifestyles and the socioeconomic developmental status of a given country. Worldwide, an estimated 19.3 million new cancer cases and almost 10.0 million cancer deaths occurred in 2020 [3]. Based on GLOBOCAN estimation, the annual global cancer burden is expected to be 28.4 million cases in 2040, a 47% rise from 2020, with a larger increase in demographically transitioning countries (64% to 95%) versus transitioned (32% to 56%) countries due to demographic changes [3].

Cancer incidence in Sub-Saharan Africa (SSA) is on the rise, caused by rapid population growth, improved life expectancy and the adoption of unhealthy lifestyles [4]. In SSA, a total of 801,392 cases were estimated to occur in 2020 [3]. According to United Nations estimates, the population of the continent will double from 1.2 billion people in 2015 to 2.5 billion by 2050 [5]. Cancer currently is a low public health priority in Africa. This is due to limited health resources and other public health issues, mainly infection-related, which are perceived as more pressing, such as malaria, tuberculosis and acquired immune deficiency syndrome [6]. Cancer treatment options in most SSA countries are sparse, and even when available, they are mostly insufficient to serve patients’ needs [7].

Similar to other SSA countries, the incidence of cancer is rising in East Africa. Based on the GLOBOCAN 2020 report, 331,233 new cases of cancer occurred in this region of Africa. Based on the same report, prostate cancer, Kaposi sarcoma and colorectal cancer were the most common types of cancer in men, while cervical, breast and colorectal cancers were the most common types of cancer in women [3].

Based on regional and global trends, cancer incidence in Somaliland is expected to increase. But, to the best of our knowledge, there is no single published data on the incidence and patterns of cancer in Somaliland. Additionally, there is no cancer registry or National Cancer Control Plan (NCCP). Until recently, cancer treatment was limited to surgery only. Patients were referred abroad for chemotherapy and radiotherapy. In July 2022, a medical oncology service was started in Needle Hospital, Hargeisa, Somaliland. This cancer clinic serves cancer patients from the whole of Somaliland and some patients from neighbouring countries. To address the scarcity of cancer data in Somaliland, we investigated the pattern of cancer cases seen at the hospital’s cancer clinic during its first year of operation. This study determines patterns of cancer with respect to patients’ regions, age, gender, comorbidities, site and subsites, histology and stage of cancer. We believe that the results of this study will help to fill the gap in data, create awareness of the magnitude of cancer in Somaliland and guide cancer control programs and policymaking.

## Methods

### Study setting

Needle Hospital, a multi-speciality hospital established in 2013, is located in Hargeisa, Somaliland. Services at Needle Hospital include pathology, internal medicine, head and neck surgery, radiology, paediatrics and child health, a laboratory and pharmacy. It is the first hospital to start pathology services in Somaliland. In July 2022, Needle Hospital established a cancer clinic. It has a centre dedicated to cancer care only, with a patient evaluation room, male and female wards, daycare rooms and a room dedicated to oncology emergencies. It also has its own oncology pharmacy and laboratory. Services provided in the centre include medical oncology for all solid tumours, palliative care and follow-up. When radiotherapy is indicated, patients are referred to radiotherapy centres abroad [8].

### Study design and population

The study used a facility-based retrospective study design. The data were extracted from a hospital-based patient registration file and charts. All patients who were evaluated in the cancer clinic from July 2022 to June 2023 were the source population, and patients with pathology-confirmed cancer were the study population. Patients whose data is missing, whose cancer is not confirmed with fine needle aspiration cytology or biopsy or who are found to have no cancer after evaluation were excluded from the study.

### Sample size determination and sampling techniques

We used total population sampling because the population size is relatively small. So all pathologically confirmed cancer patients evaluated in the cancer clinic from 1 July 2022, to 30 June 2023, were included in this study.

### Data collection tool and procedure

Records of patient information were directly extracted from hospital-based cancer patients’ registration files and charts. The variables include the age of the patient, gender, region, comorbidities, anatomical site and subsites, histology and stage of the cancer.

### Data processing and analysis

The data were coded, entered and cleaned using Excel. Then, it was exported to Statistical Package for the Social Sciences version 23 for analysis. Descriptive statistical analysis, including simple frequencies, measures of central tendency and measures of dispersion, was used to describe the data. The data were described using different statistical methods like mean, standard deviation and others.

## Results

### Socio-demographic characteristics

In the first 12 months of service, 261 patients were evaluated in the cancer clinic. Twenty-nine patients were excluded either because they had no histologic confirmation of cancer or were deemed not to have cancer. This resulted in a final sample size of 232. The median age of the cancer cases at the time of evaluation was 60 years. More than half (56.5%) of the cancer patients were female, and the remaining (43.5%) were male, with a female-to-male ratio of 1.3:1. The majority (66.8%) of the patients were from the Morodijeh region, followed by the Togdher (15.1%) and Awdal (5.2%) regions of Somaliland (Table 1).

### Cancer related characteristics

**Figure 1.**
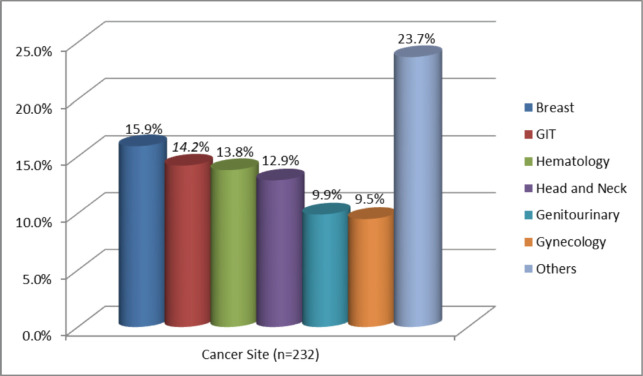
The site of the cancers from patients who were evaluated at the Needle Hospital cancer clinic in Hargeisa, Somaliland, from July 2022 to June 2023. Others: Cancer sites that are not listed here that were less frequent.

Breast (15.9%), gastrointestinal tract (GIT) (14.2%) and haematology (13.8%) were the three most common sites of cancer among the study population. Anatomical subsites that were the most common were breast, followed by oesophagus and prostate cancers (Figure 1, Table 2). Of the study participants, 24.1% had at least one comorbidity in addition to cancer. Hypertension and diabetes mellitus were the most common comorbidities, affecting 15.5% and 7.6% of participants, respectively.

**Figure 2.**
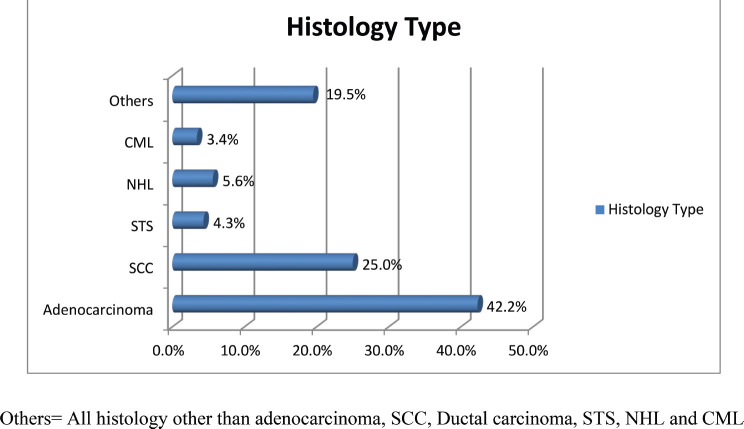
The histologic type of cancer from patients who were evaluated at the Needle Hospital cancer clinic in Hargeisa, Somaliland, from July 2022 to June 2023. Others = All histology other than adenocarcinoma, SCC, Ductal carcinoma, STS, NHL and CML.

**Figure 3.**
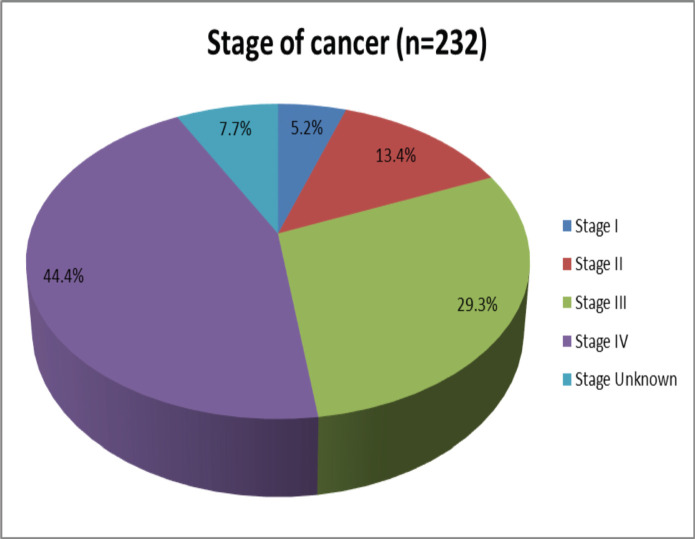
The stage of cancer from patients who were evaluated at the Needle Hospital cancer clinic in Hargeisa, Somaliland, from July 2022 to June 2023.

On histologic examination, adenocarcinoma (42.2%) was the leading histology type, followed by squamous cell carcinoma (SCC) (25.0%). A substantial number of patients presented at stage IV (44.4%), while only 18.6% of the patients presented at stages I and II combined (Figures 2 and 3, Table 3).

### Gender distribution

Among male patients, haematologic cancer (22.8%), genitourinary cancer (20.8%) and GIT cancer (17.8%) were the leading sites of cancers, while among female patients, breast cancer (28.2%), gynaecologic cancer (16.8%) and GIT cancer (11.5%) were common.

The leading cancer sub-sites among males were prostate cancer (16.8%), esophageal cancer (8.9%) and NPC (7.9%). Among females, the three leading cancers were breast cancer (28.2%), ovarian cancer (9.2%) and thyroid cancer (7.6%) (Table 4). Of the 131 female cancer patients, 22 were diagnosed with gynaecological cancers. Ovarian cancer was the most common gynaecological malignancy, with 12 cases, followed by uterine cancer with 7 cases. Ninety-two percent (92%) of ovarian cancers were adenocarcinoma by histology. The observed prevalence of cervical cancer in this study was low. This is likely due to patients with cervical cancer being directly referred to gynaecologic oncologists or radiation oncology centres for definitive treatment, bypassing the need for initial evaluation or management at the Needle cancer clinic.

## Discussion

Based on the global cancer burden estimation for 2040, low- and middle-income countries (LMICs) are expected to have a striking relative increase in the magnitude of new cancer cases [3]. The GLOBOCAN 2020 report estimated that 10,134 new cancer cases were diagnosed in Somalia, with breast and cervical cancer being the most common types. In line with this report, the findings of this first study on the patterns of cancer in Somaliland indicate that cancer is one of the major emerging health problems. In 1 year, 232 cancer patients were evaluated in the Needle Hospital cancer clinic, with the median age of the patients being 60 years. This is higher than the median age of patients in two reports in Somalia, which were 53.4 and 51.1 years [9, 10]. This could be due to women with cervical cancer, who are generally younger at diagnosis are not included in this analysis. More than half (56.5%) of the patients in this study were female, with a female-to-male ratio of 1.3:1. Similar findings were reported from Ethiopia. Two studies, the Addis Ababa city population-based cancer registry and Wolaita Sodo University, showed the majority of patients were female (67% and 62.8%, respectively) [11, 12]. A recently published paper showed cancer ranks among the top three causes of premature mortality in women in almost all countries, and it is less amenable to primary prevention in women than in men [13]. The majority (66.8%) of the patients came from the Morodijeh region; this could be due to the geographical proximity of the region to the cancer clinic, the fact that this region is more populated, and it is where the capital city of Somaliland is located [14].

Based on anatomic sub-sites, the most common cancers in this study population were breast, esophageal and prostate cancers. Breast cancer accounted for 15.9% of all cases, and this is similar to the GLOBOCAN 2020 Somalia report [15]. Breast cancer was also the leading cancer in the first report of the Addis Ababa city population-based cancer registry [11]. From the first results on cancer incidence in Khartoum, Sudan, breast cancer was the leading type [16]. Breast cancer is the leading cause of cancer deaths among women, and it is disproportionately affecting LMICs. To improve breast cancer outcomes in Somaliland, we recommended implementing the WHO Global Breast Cancer Initiative (health promotion for early detection, timely diagnosis and comprehensive breast cancer management) [17].

Different from other regional reports [12, 18] and the GLOBOCAN 2020 Africa report [19], esophageal cancer was the second most common anatomic site in this study, accounting for 8.2% of the cases. In studies from two university hospitals in Ethiopia, Wolaital Sodo University Hospital and University of Gonder Hospital, esophageal cancer was not among the 10 most common cancer types [12, 18]. However, two reports from Somalia showed esophageal cancer was the leading type of cancer [9, 10]. In the first study on the frequency and distribution of the pathology results of 403 cancer cases diagnosed between 2016 and 2017 at the Somali Mogadishu Turkey Education and Research Hospital and in the oncology department at Uniso Hospital/Somalia University, oesophageal cancer was the leading cancer, accounting for 32.3% [9]. In the second study on the incidence and distribution of cancer at a tertiary care hospital in Somalia (Somalia Turkey Recep Tayyip Erdogan Education and Research Hospital) over 3 years, 1,306 patients were involved in the study and esophageal cancer was the leading cancer, accounting for 21.7% of cases [10]. Even though SCC of the esophagus has become less common in North America and Western Europe in recent decades and now accounts for less than 30% of all esophageal cancers, 84% of the esophageal cancers in this study were SCC in histology [20]. The reason why oesophageal cancer is common in our study may be multifactorial, including environmental and dietary habits such as ingesting hot meals. However, the aetiology and clinicopathology of esophageal cancer in this population should be investigated in order to institute adequate preventive and therapeutic measures.

This study also showed most patients presented at advanced stages; stage IV and stage III accounted for 44.4% and 29.3%, respectively. This is similar to a retrospective analysis of the pattern of cancer care and radiotherapy in Ethiopia from 2015 to 2018 on 1823 cancer patients treated with cobalt radiotherapy in Tikur Anbesa Hospital, where stage III and IV combined accounted for 73.5% [21]. Another study on stage at diagnosis, clinicopathological and treatment patterns of breast cancer in northwestern Tanzania showed a similar trend in cancer stage, where stage III and IV combined accounted for 84.4% of all breast cancer patients [22].

To decrease the incidence of cancer, diagnose it at an early stage and improve cancer care services that can deliver comprehensive multi-modality treatment, we recommend developing NCCP, improving cancer literacy by conducting community cancer awareness campaigns on cancer risk factors and symptoms and implementing a cancer screening program. We also recommend establishing a population-based or national cancer registry. Finally, we suggest a cancer policy that involves an emphasis on evidence-based cancer prevention, early presentation, timely diagnosis and comprehensive timely treatment.

## Conclusion

This initial study on the pattern of cancer in Somaliland confirms that cancer is a major emerging health concern. In 1 year, 232 cancer patients were evaluated in the Needle Hospital cancer clinic. The median age of the patients was 60 years, and 56.5% of the patients were female. The majority of the patients were from the Morodijeh region of Somaliland, followed by the Togdher and Awdal regions. Most patients presented at an advanced stage. Breast, esophageal and prostate cancers were the most commonly diagnosed cancers. Esophageal cancer being a common finding, is disparate, so a study to investigate the aetiology and biology of oesophageal cancer is recommended. We recommend establishing an NCCP which will include setting up a national cancer registry and developing workforce and research capacity. Finally, to improve cancer outcomes, we recommend capacity building in diagnostic and treatment facilities alongside regional and international collaboration.

## List of abbreviations

CML, Chronic myelogenous leukemia; DM, Diabetes mellitus; GIT, Gastrointestinal tract; GLOBOCAN, Global Cancer Observatory; NCCP, National Cancer Control Plan; NHL, Non-Hodgkin’s lymphoma; NPC, Nasopharyngeal carcinoma; SCC, Squamous cell carcinoma; SPSS, Statistical Package for the Social Sciences; STS, Soft tissue sarcoma; SSA, Sub-Saharan African; UN, United Nations; WHO, World Health Organisation.

## Conflicts of interest

The authors declared no potential conflicts of interest with respect to the research, authorship and/or publication of this article.

## Funding

There was no financial fund for this research.

## Data availability

Data used for this research will be available upon request.

## Ethical approval and consent to participate

Ethical clearance for the study was obtained from the Republic of Somaliland Ministry of Health Development (MOHD/DG/1181/2023). To ensure the confidentiality of the study participants, we avoided all possible personal identifiers in this study. This research was carried out in conformity with the Declaration of Helsinki.

## Figures and Tables

**Table 1.** Sociodemographic characteristics of cancer patients who were evaluated at the Needle Hospital cancer clinic in Hargeisa, Somaliland, from July 2022 to June 2023.

Variables		Frequency	Percent (%)
Gender	Male	101	43.5
	Female	131	56.5
Region	Morodijeh	155	66.8
	Togdher	35	15.1
	Awdal	12	5.2
	Sanaag	10	4.3
	Sahil	5	2.2
	Sool	3	1.3
	Others^*^	12	5.1

* Others = Djibouti, Ethiopia

**Table 2.** The sub-sites of cancer from patients who were evaluated at the Needle Hospital cancer clinic in Hargeisa, Somaliland, from July 2022 to June 2023.

R.NO	Cancer subsite	Frequency	Percent (%)
1	Breast	37	15.9
2	Esophageal	19	8.2
3	Prostate	17	7.3
4	Nasopharyngeal carcinoma	13	5.6
5	Thyroid	12	5.2
6	Ovarian	12	5.2
7	Lung	10	4.3
8	Liver	8	3.5
9	Soft tissue sarcoma	7	3.0
10	Skin	5	2.2
11	Others*	92	39.6

Others*: = Cancer subsites that are not listed here that were less frequent

**Table 3.** The common site and their respective histology types from patients who were evaluated at the Needle Hospital cancer clinic in Hargeisa, Somaliland, from July 2022 to June 2023.

Cancer sites	Total patients	Histology	
		Type	Percent (%)
Breast	37	Ductal	97.3
		Lobular	2.7
GIT	33	SCC	60.6
		Adenocarcinoma	36.4
		Others *	3.0
Hematology	32	NHL	40.6
		CML	25.0
		Others ^*^	34.4
Head and neck cancers	30	SCC	90.0
		Adenocarcinoma	3.3
		Others *	6.7

Others*: = All histology other than adenocarcinoma, SCC, Ductal carcinoma, STS, NHL and CML

**Table 4.** Gender-based cancer subsites from patients who were evaluated at the Needle Hospital cancer clinic in Hargeisa, Somaliland, from July 2022 to June 2023.

Variables			Percent (%)
Subsites of cancers	Males	Prostate cancer	16.8
		Esophageal cancer	8.9
		NPC	7.9
		Thyroid cancer	2.0
		Others	64.4
	Female	Breast cancer	28.2
		Ovarian cancer	9.2
		Thyroid cancer	7.6
		Esophageal cancer	7.6
		Others	47.4
